# Letter to the editor: commentary on “Prevalence of colonoscopy-related adverse events in older adults aged over 65 years: a systematic review and meta-analysis”

**DOI:** 10.1097/JS9.0000000000002905

**Published:** 2025-07-07

**Authors:** Dexin Wang, Yongping Mu, Jinzhong Yu

**Affiliations:** a Shuguang Hospital Affiliated to Shanghai University of Traditional Chinese Medicine (TCM), Institute of Liver Diseases, Cell biology laboratory, Shanghai University of TCM, Key Laboratory of Liver and Kidney Disease of the Ministry of Education, Clinical Key Laboratory of TCM of Shanghai, Shanghai, China; b Department of Gastroenterology, Shuguang Hospital Affiliated to Shanghai University of Traditional Chinese Medicine, Shanghai, China

*Dear Editor,*

We recently read with great enthusiasm the systematic review and meta-analysis published by Lu et al^[1,2]^, and we sincerely congratulate the authors on their outstanding work, which deeply explores the prevalence of colonoscopy-related adverse events in older adults aged over 65 years, providing valuable insights into this field. Colorectal cancer (CRC) is the second leading cause of cancer-related deaths globally and ranks third in incidence. The rising incidence and mortality rates pose significant challenges to global health^[3]^. As the gold standard for CRC diagnosis, colonoscopy plays a crucial role in the early screening and management of this disease^[4]^. With economic development and the accelerating trend of population aging, an increasing number of older adults require colonoscopy^[5]^.

Although this study fills a gap in the literature, there are still several points that merit further exploration and discussion to strengthen future research in this area.

First, according to the authors’ findings, the prevalence of colonoscopy-related perforation and bleeding in individuals over 65 years of age is 0.078% and 0.235%, respectively. This finding is particularly striking, as the risk of perforation and bleeding in the very elderly group (aged over 80 years) is significantly higher than that in the younger elderly group (aged 65–80 years). Additionally, the relatively high incidence of cardiopulmonary adverse reactions related to colonoscopy is also an issue that public health must address. The authors’ detailed exploration of these unconfirmed issues in the meta-analysis, as well as their presentation of confirmed viewpoints in clinical practice, is commendable.

However, it is worth mentioning that the quantity and scope of existing studies somewhat limit the applicability of the conclusions. While most studies included in the review have large sample sizes, four studies have samples of fewer than 10 000 participants, which is significantly lower than the average sample size of other studies (73,436 participants). This small sample size may introduce data bias, making the conclusions less reflective of the true situation of colonoscopy among older adults worldwide. Notably, current known studies are mostly concentrated in countries with high social development indices (such as the United States, Japan, Canada, South Korea, France, and Sweden), where good economic and medical conditions enable individuals aged 65 years and over to access high-quality screening and treatment. This concentration not only indicates that colorectal screening and cancer prevention require substantial funding and expertise but may also lead to the neglect of other regions. As a result, the study results may only reflect the characteristics of areas where colonoscopy is relatively safe and may not represent the situation of older adults globally.

Furthermore, the age stratification in the studies could be more detailed. Currently, analyses have not been conducted for other age thresholds, such as 70 or 75 years, which may fail to comprehensively reflect the risk differences that older adults face during colonoscopy. Additionally, studies often lack specific details about intraoperative anesthesia, the proficiency of endoscopists, and the endoscopic equipment used. The absence of this information hinders a comprehensive assessment of the risks associated with the procedure. In terms of subgroup analysis, due to the insufficient data included in the studies, more in-depth analyses of individual parameters that may affect adverse events during colonoscopy, such as health status, comorbidities, and long-term medication use, have not been conducted. These factors are crucial in clinical practice for assessing patient risk, and future research should focus on these aspects to develop more personalized screening protocols. Notably, none of the included studies reported mortality data, and there was no unified definition or classification standard for cardiopulmonary adverse events within the studies, which could impact the results. Clearly, these issues primarily stem from a deficiency in baseline data collection by endoscopists, and they may also be related to the division of endoscopy information systems from patient electronic medical record systems in many healthcare institutions, leading to a lack of baseline information—this is a common issue in endoscopic research.

Therefore, we call for more international collaboration and multicenter studies to encompass a broader population base, particularly in low- and middle-income countries. Only by obtaining more comprehensive research data can we more accurately understand the significance and impact of colonoscopy in the health care of older adults globally. This will be crucial for increasing screening rates and cancer management among elderly patients in these regions. With these thoughts in mind, let us work together to promote the global dissemination of CRC primary and secondary prevention measures, including those involving colonoscopy, to provide safer and more effective medical services for all older patients.

## Data Availability

No.
